# Defining a Domain-Specific Language for Behavior Verification of Cyber–Physical Applications

**DOI:** 10.3390/s25216720

**Published:** 2025-11-03

**Authors:** Konstantinos Panayiotou, Emmanouil Tsardoulias, Theodoros Tsampouris, Andreas L. Symeonidis

**Affiliations:** School of Electrical and Computer Engineering, Aristotle University of Thessaloniki, 54636 Thessaloniki, Greece; etsardou@ece.auth.gr (E.T.); ttsampob@ece.auth.gr (T.T.); symeonid@ece.auth.gr (A.L.S.)

**Keywords:** behavior verification, low-code development, domain-specific languages, model-driven development, internet-of-things, cyber–physical systems

## Abstract

A common problem in the development of Internet-of-Things (IoT) and cyber–physical system (CPS) applications is the complexity of these domains, due to their hybrid and distributed nature at multiple layers (hardware, network, communication, frameworks, etc.). This complexity often leads to implementation errors, some of which result in undesired states of the application and/or the system. The current work focuses on low-code development of behavior verification processes for IoT and CPS applications, in order to raise productivity, minimize risks (due to errors) and enable access to a wider range of end-users to create and verify applications for state-of-the-art domains, such as smart home and smart industry. Model-Driven Development (MDD) approaches are employed for the implementation of a Domain-Specific Language (DSL) that enables the evaluation of IoT and CPS applications, among others. The proposed methodology automates the development of behavior verification processes, allowing domain experts to focus on the real problem, instead of struggling with technical and technological breaches. Through comparative scenario-based analysis and 43 detailed use cases, we illustrate how the proposed methodology automates the development of behavior verification processes, allowing end-users to focus on the verification definition, instead of technical and technological intricacies.

## 1. Introduction

The rapid advancement of information technology, particularly through the integration of the Internet of Things (IoT), cyber–physical systems (CPS), and smart infrastructures, has played a significant role in digitization across various sectors. This transformation is particularly evident in the design and development of smart cities and smart grids, which aim to enhance the quality of urban life and streamline essential services. The integration of IoT and Artificial Intelligence (AI) is pivotal in this context, as it facilitates improved management and sustainability, ultimately benefiting citizens’ daily lives. In addition, the IoT facilitates the creation of ecosystems of heterogeneous and distributed systems and services that enable access to functionality and data provided by physical devices equipped with electronics, sensors and actuators, and embedded software, the so-called smart objects [1,2]. In turn, smart objects can foster important changes in our lives as they are increasingly pervading the environments we live in. However, while research on IoT has devoted many efforts to the technological aspects characterizing smart objects, few social and practical benefits have emerged so far. Programming the behavior of smart objects is currently a task assigned to professional engineers and domain experts only, as it requires excessive knowledge in various scientific and engineering fields, such as programming languages, web technologies, networks and communication, cloud technologies, and firmware development (apart from domain expertise).

It should also be mentioned that many domains are adopting IoT technologies, such as healthcare, industry, smart cities, agriculture, transportation and critical infrastructures (e.g., energy, water and telecommunication). The explosive growth of smartphones and tablets brought the number of devices connected to the Internet to 12.5 billion in 2010, which is almost 2 (1.84) connected devices per person [3]. Research studies show that the number of connected devices in 2020 was 18 billion [4], while the number of devices is expected to exceed 25.44 billion by 2030 [5]. Major industry verticals with currently more than 100 million connected IoT devices are electricity, gas, steam & A/C, water supply & waste management, retail & wholesale, and transportation & storage. The most important use case for IoT devices in the consumer segment is personal devices such as smartphones and tablets [4]. Other use cases with more than one billion IoT devices by 2030 are autonomous vehicles, IT infrastructures, asset tracking & monitoring, and smart grid.

We argue that in order to fully unleash the potential capabilities of IoT and CPS, it is important to facilitate application development from an end-user perspective; the number of potential designs of connected smart objects is endless and will boost IoT technology to new frontiers. However, moving from an industry-driven (the smart objects manufacturers) to an end-user perspective is a more challenging task [6]. It is common knowledge that developing IoT applications requires knowledge in various layers (and software)-related disciplines: low-level hardware details for connecting sensors and actuators into embedded and IoT devices, firmware development for controlling connected sensors and actuators, networking and web technologies for connecting devices into IoT middlewares, gateways and platforms, data manipulation, data analysis and software development. In [7], the authors note that the heterogeneity of IoT devices is an obstacle for developers in terms of developing applications and sharing information, while in [8], the authors present a list of challenges and opportunities in a generic format with regard to the development of IoT applications. In fact, many researchers classify IoT developers into three main categories [9]: (1) Technology experts and scientists, (2) General application developers, and (3) Domain experts or end-users.

Moreover, the growing number of software-defined IoT platforms and the heterogeneity of physical objects make application development a difficult and time-consuming task, even for expert developers. A technological approach currently being able to solve such problems is Model-driven Development (MDD), because it can provide high-level concepts for the description of the under study domain [10]. MDD is a software development methodology that allows users to build complex applications through simplified abstractions of pre-built components [11]. On the other hand, MDE suggests a development process based on model creation and manipulation, via the utilization of Model-to-Text (M2T) and Model-to-Model (M2M) transformations [12].

In order to enable rapid development of IoT and CPS systems and overcome the aforementioned challenges, the low-code/no-code paradigm and specifically DSLs seem to be a promising alternative. In fact, in their study [13], D. Alulema et al. mention that their DSL allows developers to define a new architecture without extensive knowledge of the hardware and software design of IoT systems. Overall, DSLs are languages tailored to a specific application domain [14] and therefore have the potential to reduce development complexity by raising the abstraction level. According to the application domain and the targeted group of users, different notations (textual, graphical, tabular) are used. Nowadays, DSL engineering approaches are utilized for the development of low-code [15] and no-code [16] solutions (e.g., frameworks and platforms) and enable access to a wide range of users and application domains. These solutions provide an abstraction layer and user-oriented semantics for the description of various aspects of the application domain(s) [17]. Furthermore, they implement syntactic and semantic validation so that the user can ensure that certain properties of the understudy domain/system/application hold true via a set of meta-models and constraints that describe the subject domain. Additionally, model transformation and model interpretation processes are often delivered to enable automated generation and deployment of software artifacts from input DSL models [12].

In this work, we explore the difficulties of verifying the behavior of IoT-enabled CPS applications and we propose a low-code approach based on Domain-Specific Languages, in a way that enables rapid development of behavior verification scenarios [18] in a context-aware (topology of the environment) and protocol-agnostic architecture (supports common communication protocols and patterns for edge and mixed edge-cloud environments). Our approach assumes already connected entities to a message/event-driven architecture, providing common communication protocols, such as MQTT, CoAP, AMQP and Redis. These entities can be physical devices, such as sensors, actuators, and mobile robots, or virtual, such as software-defined agents, monitoring, storage software, etc. This paper presents a novel methodology that automates the development of behavior verification processes. Through comparative scenario-based analysis and 43 detailed use cases, we demonstrate how this approach empowers end-users to concentrate on core problem-solving, effectively eliminating the need to contend with technical and technological complexities. Our methodology’s versatility extends to both real and virtual CPS, and it is directly applicable to Digital Twin systems [19], a domain where establishing trust through robust verification and validation frameworks is an active and critical research challenge [20]. Finally, we evaluate our approach against two research questions:

- RQ-1: Can we accelerate the development of the behavior verification process for Cyber–Physical and Digital Twin applications?
- RQ-2: Does our approach need minimum domain knowledge, and is it framework-, protocol- and device-agnostic?

### 1.1. State of the Art

Comparative studies show that domain-specific languages (DSLs) offer significant advantages over general-purpose languages (GPLs) in the design and verification of cyber–physical systems (CPS). Empirical research demonstrates that developers using DSLs achieve higher accuracy and efficiency in program comprehension tasks compared to those using GPLs, both with and without integrated development environments, validating long-held beliefs about the benefits of DSLs for domain-relevant tasks [21,22]. DSLs enable higher-level abstractions tailored to specific CPS domains, reducing complexity and improving expressiveness, which facilitates more effective modeling, verification, and deployment [14,23,24]. In contrast, GPLs provide broader applicability but often require more boilerplate and domain-specific adaptation, which can hinder productivity and increase the risk of errors in CPS contexts. Furthermore, DSLs can encapsulate domain knowledge and constraints, supporting verification and runtime monitoring, as seen in languages like RML for runtime verification [25].

Latest secondary studies, which include systematic literature reviews and comparative studies, are presented in [26,27]. The authors discuss the state of the art on existing approaches supporting the development of IoT systems and focus on DSLs and tools available in the MDE field and the emergent low-code development platforms covering different aspects of the IoT domain. In their work [27], Sadik Arslan et al. analyzed 32 different DSLs that have been designed for IoT software development in terms of a set of requirements (language definition, language features, and tool support). Moreover, the authors perform an analysis based on the non-functional properties of the DSLs, such as performance, security, safety, reliability, availability, planability, portability, and resource consumption. The results of these studies can serve as a guide for using and developing DSLs for the IoT domain and for selecting the most appropriate modeling language for dedicated development purposes (e.g., device/system/application-level development).

While numerous DSLs exist within the IoT and CPS landscape, they can be broadly categorized according to their primary objectives. A significant portion focuses on application development and system modeling, aiming to simplify the definition and creation of IoT systems. For instance, ThingML [28,29], FRASAD [30], and SmartHomeML [31] provide abstractions for generating application code, managing device interactions, and defining rule-based logic. Similarly, Triton [32] reduces boilerplate for developers by managing tasks and constraints at the code level. Although these tools are powerful for system construction, they do not offer dedicated mechanisms that enable end-users to formally specify and verify the emergent behavior of the complete, deployed system.

Another category of tools/DSLs focuses on the resource-oriented monitoring of CPSs (involving memory, computational, and networking resources). Mancilla’s work [33] addresses the automatic generation of industrial CPSs from standardized descriptions of industrial processes, incorporating runtime supervision and performance assessment at the resource level. Similarly, Aguzzi’s work [34] enables the automatic generation, configuration, and supervision of industrial CPSs, in which users define resource-oriented verification parameters that an ML classifier uses to detect anomalies. Although these approaches allow dynamic (real-time) inspection of a system, they remain limited to resource checking and are therefore not generic enough to support user-defined validation rules.

Moreover, another category of DSLs incorporates formal methods for system specification, such as the work by Andova et al. [35], which transforms models into Labeled Transition Systems (LTS) for verification. Similarly, Monitor-IoT [36] is an MDE tool and language that, apart from defining physical and digital entities, supports the specification of monitored properties, enabling the structural design of the monitoring process without behaviorally verifying the actual CPS. Another formal modeling language and toolchain is aDSL [37], which, although it supports the modeling of CPSs and their requirements, allows only static system verification (at the design phase). Similarly, other recent work focuses on ensuring properties like consistency and availability at the early design stages of complex distributed systems, such as those involving Edge AI [38], in contrast to our proposed language, which operates during runtime.

Finally, there are formal approaches to CPS verification, such as the ETL language [39], which corresponds to the FORM-L language [40] of the MODELICA framework [41]. These are quite similar to our approach, since ETL offers a close-to-natural-language syntax for expressing high-level requirements and constraints. Nevertheless, the drawback of such tools is that they are technology- or vendor-specific, as they operate only within the limits of their supported toolsets. In contrast, our approach is technology-agnostic; through appropriate transformation processes, GoalDSL validations can be applied to any broker-based system.

To summarize, our work addresses a distinct gap: rapid development of goal-driven, dynamic, and technology-agnostic behavior verification processes for IoT and CPS applications. Instead of focusing on how to construct an application, our approach focuses on specifying and verifying ‘does the deployed application behave as expected?’. This shifts the focus from implementation details to observable outcomes in a protocol-agnostic architecture, which is critical for complex CPS and Digital Twin environments. The following table systematically compares our approach with the state of the art based on key characteristics.

As summarized in Table 1, existing approaches predominantly cater to developers and focus on the generation and modeling of IoT/CPS applications. They employ paradigms such as rule-based or state-based programming to construct system logic. While some offer design-time verification through model checking or formal methods, they lack a dedicated, user-friendly abstraction for defining and testing high-level behavioral goals at runtime.

Our work provides a distinct contribution by addressing the need for a domain-specific language (DSL) for behavior verification of IoT and CPS applications. Its novelty lies in empowering domain experts—not just developers—to specify expected outcomes (i.e., the “what”) without delving into the technical implementation (the “how”). By being technology- and protocol-agnostic, our approach is uniquely suited for verifying the behavior of heterogeneous systems, including Digital Twins, thereby addressing the critical research questions of accelerating the verification process (RQ-1) and minimizing prerequisite technical knowledge (RQ-2).

### 1.2. Outline of the Paper

The rest of the paper is structured as follows: Section 2 discusses our approach and the proposed DSL; Section 3 includes our experimental validation methodology, along with discussions, limitations, and threats to validity. Finally, the current study is concluded, and future work is discussed in Section 4.

## 2. Methodology

As already discussed in the previous section, many low-code and DSL approaches related to the IoT and CPS domains exist. However, most of them focus on serving the requested functionality at the “thing” or the application layers [26,27], while none of them addresses whether the requirements of such “smart” systems are satisfied. It is common knowledge in the intelligent systems literature that the executed behavior may not result in satisfying the planned goal. The current study focuses on the use of Domain-Specific Languages for the description of verification processes for IoT-enabled CPS, with an emphasis on smart environments and the automated generation of the software artifacts involved in the verification processes. Our model-driven approach practically automates the verification process of the expected behavior of the system under development.

The proposed DSL, namely GoalDSL, focuses on the behavior-level verification of IoT-enabled Cyber-Physical applications based on a goal-driven approach. The general idea is that goal-driven rules can be defined for entities (smart objects, virtual artifacts, etc.) in a CPS or a digital twin, focusing on the context of smart environments. For example, a goal may define a rule to wait until an entity changes its state (changes in attributes). To expand this idea to the context of IoT-enabled CPS, it is useful to define environment-related rules for mobile objects, such as robots. For example, in robotics, it is common to require the definition of goals related to the pose of the robot, to follow a trajectory, to avoid an area, etc. GoalDSL provides a number of built-in domain-specific goal types, such as pose, trajectory, and area related, but it also provides generic types that directly bind to entities and apply conditions based on their state $E_{n}\left(t\right)$. The state of an Entity is defined by the values of its attributes.

Let’s consider an example of a Digital Twin of smart environments, which has a number of sensors and actuators installed in various sectors (rooms). These smart objects are connected to a communication middleware (message broker) that is commonly used in modern IoT applications, enabling them to receive and/or send messages. Message brokers are pieces of software that enable bidirectional communication between the endpoints in distributed systems via message queues [43]. Message topics are defined for communicating with smart objects, which are based on unique resource identifiers (URIs). Each resource of a smart object is considered an Entity at the application layer, which has a name, communicates via a specific topic, and has a strict data model defined by its attributes. For example, a smart object having a temperature sensor installed defines an Entity named “temperature_sensor”. Multiple entities can exist for a single smart object, such as a device that has two relays and a buzzer installed. In such cases, each sensor/actuator is defined separately.

In the aforementioned example, GoalDSL can be utilized for the behavioral verification of various automation scenarios and raise alerts when goal errors occur. This is crucial to prevent reaching undesired, faulty states in automation apps in real scenarios in an asynchronous and highly distributed system, such as in the case of smart environments and CPS in general. Our goal-driven approach allows for the definition of goals that are met based on conditional rules that apply to message topics. Conditions are usually applied to sensor messages, such as in-house temperature and humidity measurements. Finally, in the context of the current work, GoalDSL has been developed to lower development complexity and, therefore, time, allowing for the definition of ‘solution’ models using simple semantics.

The following section discusses our approach and provides in-depth information on GoalDSL for developing behavior verification processes for IoT-enabled Cyber–Physical applications. Section 2.1 presents an overview of our approach, while Section 2.2 discusses domain modeling, grammar definition, model creation, and automated software generation processes.

### 2.1. DSL Definition

**Figure 1:**
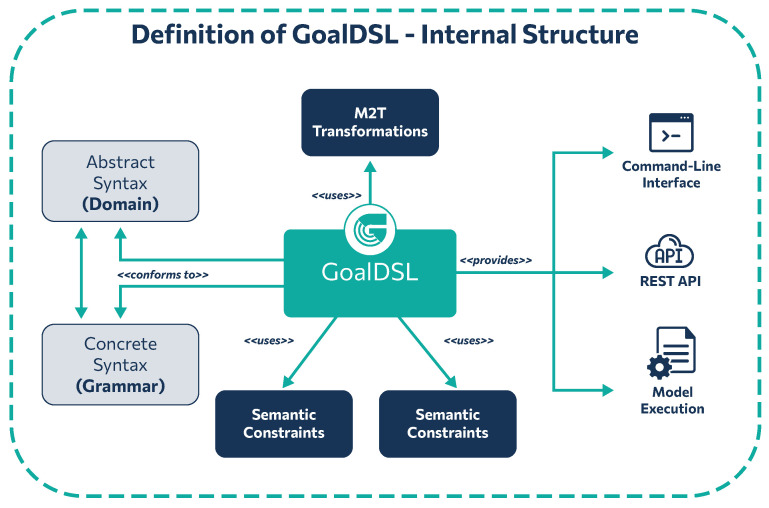
Internal architecture of the DSL integrating model validation and automated code generation processes.

GoalDSL comprises four core components: (1) the meta-model, which defines the concepts and their relations; (2) the grammar of the language for describing instance models; (3) the model validator, which validates instance models against the meta-model and a set of constraints; and (4) the source code generators, which are responsible for transforming models into source code via the execution of a Model-to-Text transformation. Moreover, in the context of the current work, an internal DSL [44] was developed in the Python programming language, namely Goalee (Goalee Internal DSL: https://github.com/robotics-4-all/goalee, accessed on 21 October 2025) that implements several concepts of GoalDSL and is used by the code generator to produce the source code from a given input model. Goalee can also be used as a standalone Python library for implementing goal-driven scenarios for applications. Figure 1 presents the internal structure of the DSL. Validation is performed on input instance models before the generation of the relevant behavior verification processes with Goalee.

In addition, GoalDSL provides a command-line interface (CLI) for performing actions on input models, such as verification, code generation and execution, and a REST API for remote access to the provided functionality to support deployment in cloud and on-premise infrastructures, as well as integration with low-code platforms. Table 2 shows the API endpoints, their URIs, available HTTP verbs, and a description of the functionality provided.

The proposed DSL and the provided tools are hosted on a github repository (https://github.com/robotics-4-all/goal-dsl, accessed on 21 October 2025) and released under the MIT license, offered as open access software. Furthermore, the project is well documented for the user to start creating and deploying behavior verification processes in just a few minutes.

### 2.2. Abstract & Concrete Syntax

This Section discusses domain meta-modeling (abstract syntax) and grammar definition (concrete syntax) of GoalDSL. The general idea behind our approach is that one may define rules applied to entities within a smart environment, such as sensors, actuators, robots or any other software-defined (virtual) processes. For example, a simple goal may define a rule to wait until the state of the thermostat changes, or until a certain condition of its state is met (e.g., the temperature is higher than 30 degrees).

**Figure 2:**
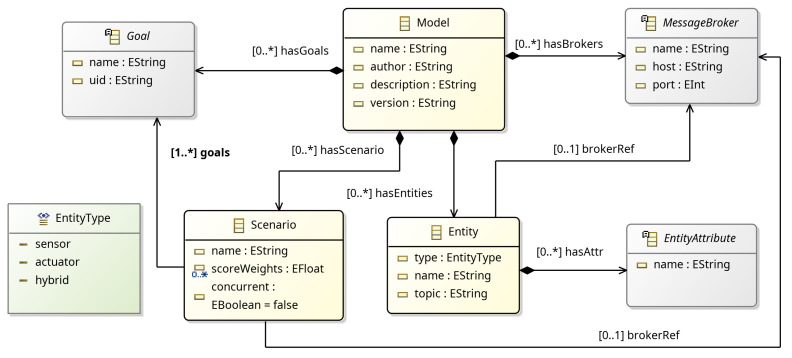
Root meta-model of GoalDSL that includes the core concepts of the DSL.

The core concepts of the meta-model, along with their relations, are depicted in Figure 2. Entities are connected to a MessageBroker and have a set of Attributes, which define the data model and its current state. Next, a Scenario defines the behavior verification process that includes a number of Goals with optional score weights, used for calculating the final score of the under study scenario, a reference to a MessageBroker for explicit definition of a single broker to use (overwrites the relevant reference from Entity definitions) and can be set to run in sequential or concurrent mode.

The grammar of the DSL is a Parsing Expression Grammar (PEG) [45], a type of analytic formal grammar. It uniquely supports the modular development of models by allowing definitions of entities, goals, scenarios, and brokers to be split into separate files and reused via a simple “import” statement. This is very useful due to the conceptual separation between the design of the system and the definition of the automation tasks on top of that. Furthermore, in-language comments and references using fully-qualified names in dot notation are also supported.

According to the literature [46], there are two key factors that contribute to the complexity of the optimal value *S*(size) of a meta-model that lies within the range of [20, 60] concepts. Although the internal structure affects the ease of meta-modeling, even a simple count of the elements of the meta-model is an approximation for the qualitative assessment of complexity, which does not differ significantly from other metrics. Therefore, the evaluation of the meta-models is based on the simple counting of concepts defined by each meta-model. Meta-models for which *S* values of less than 20 are obtained usually refer either to subfields that are meta-modeled with minimal concepts or to the meta-modeling of concepts that are reused in other meta-models, a practice that was also followed in this study. Furthermore, according to [47], a meta-model *M* has two types of terms and two types of relationships, which contain the concepts of the sub-domain, which together form the set *C*. The set of interrelationships is denoted by *A*, while the set of referenced terms belonging to other subfields (and therefore other meta-models) is denoted by *E*. Finally, the set of references of the elements of *C* to the elements of the set *E* is denoted by *R*. Conclusively, the size *S* of the meta-models is calculated based on the following equation.

(1) $$S=|C\cup A\cup \Sigma_{i=1}^{n}\left(E_{i}\cup R_{i}\right)|$$

GoalDSL has been designed in a multi meta-model approach, and specifically implements 6 meta-models: (a) GoalDSLRootMM that defines the root concepts of the language (e.g., Scenario), (b) GoalMM that defines the goal-related concepts, (c) ConditionMM for the definition of conditional expressions in models, (d) DataTypeMM that provides in-language data types, (e) EntityMM that provides the concepts related to entity definitions, and (f) CommunicationMM that provides the communication aspects of the language, such as message broker connections. The per meta-model metrics are presented in Table 3, and the meta-model size measurements (*S*) indicate strong adoption of separation of concerns for the definition of the DSL.

Next, the most important concepts of the DSL are presented in detail, including meta-models, relevant grammar and examples.

#### 2.2.1. Entity

**Figure 3:**
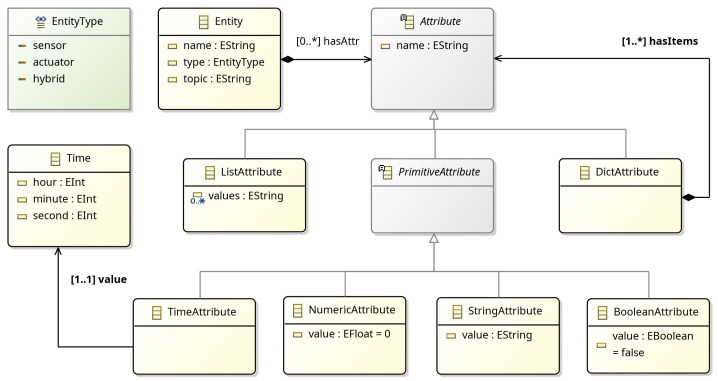
Entity meta-model.

Physical and virtual objects in a CPS are mapped to entities using the Entity meta-model in Figure 3. Entities have a variable number of attributes (Attribute) and can be used to define the data model of sensors and actuators, as well as software-defined algorithmic processes, such as an object or face detector, or a navigation stack running on a robotic platform. In addition, each Entity has a *type* property that is used to explicitly define a sensor, actuator, or a hybrid object. Notice that each Entity has its own reference to a MessageBroker; thus, the meta-model allows for communicating with entities connected to different message brokers. This allows the development of behavior verification scenarios for multi-broker architectures. The *topic* property defines the broker URI for sending and receiving messages.

The example below illustrates the definition of an Entity in GoalDSL, for a temperature sensor installed in the bedroom of a smart home, and has the temperature Attribute. The sensor is connected to the CloudBroker and messages are sent to the bedroom.temperature topic. Finally, note the use of the optional *freq* property set to the value of five (5) Hz, which is a property used to generate software for virtual entities. This functionality of the DSL (i.e., to automatically generate virtual entities from model definitions) will be discussed in more detail in Section 3.1.3.   


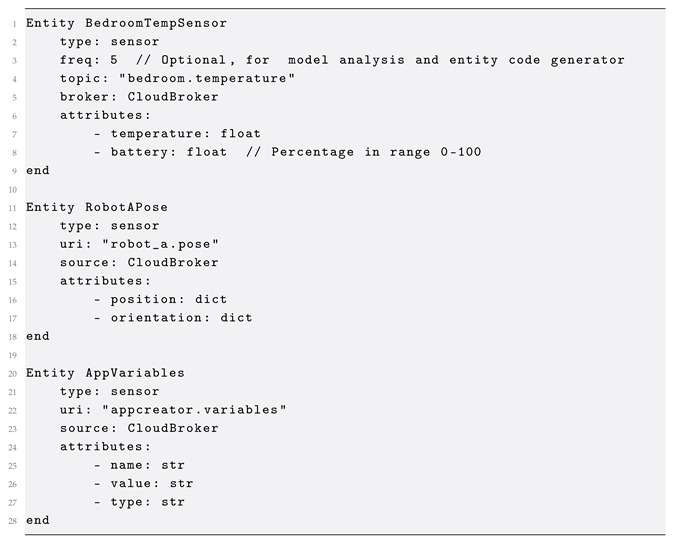


The AppVariables Entity represents a crucial sensor component designed to monitor and expose application-specific variables within the smart environment. Defined with a uri of storage.variables, this entity is configured to source its data from CloudBroker, indicating its role in accessing dynamic, in-memory data pertinent to the application’s state. Its attributes—name, value, and type, all defined as strings—enable the entity to capture the identifier, current content, and data type of each application variable, providing a flexible mechanism for integrating application logic and state into the broader automation and monitoring framework.

A significant strength of the current study lies in the extensibility of the Entity concept, which promotes interoperability with other parts of CPS. As previously discussed, entities are not limited to physical or virtual devices; they can also be defined to represent and seamlessly integrate with consumer cloud services. This capability allows our framework to connect with and leverage external functionalities such as cloud-based storage, advanced data analysis platforms, sophisticated monitoring solutions, and powerful data aggregation services, all through a common broker interface. Furthermore, this extends to defining producer external services, effectively allowing for the dynamic augmentation and enhancement of an environment’s functionalities by incorporating external data sources or computational capabilities. This broad support for diverse external services, both consumer and producer, makes our approach highly adaptable and capable of addressing complex, distributed smart environment scenarios.

#### 2.2.2. Broker

**Figure 4:**
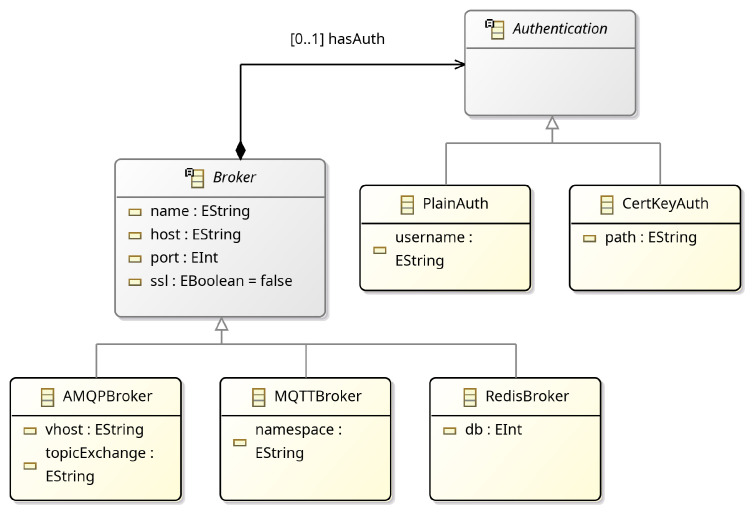
Communication (Broker) meta-model.

As earlier discussed, entities are considered connected endpoints and the MessageBroker concept defines the communication and messaging middleware. Currently, MQTT, AMQP and Redis brokers are supported in GoalDSL (Figure 4), but this can be easily extended to follow technological trends (e.g., to include Kafka). Moreover, the language currently supports plain (username, password) and client cert key authentication mechanisms for connecting to the message brokers, while it also supports platform-specific properties of brokers, such as the *vhost* and *topicExchange* for AMQP brokers. A common optional parameter across all message broker classes is ssl, which facilitates secure SSL/TLS communication.

Our approach offers significant flexibility by enabling interaction with Entities connected to diverse message brokers. This allows for the creation of verification processes that can seamlessly interact with multiple environments. For instance, consider the following example of defining an MQTT broker within GoalDSL, configured to use a plain authentication mechanism for connecting to a cloud broker:   


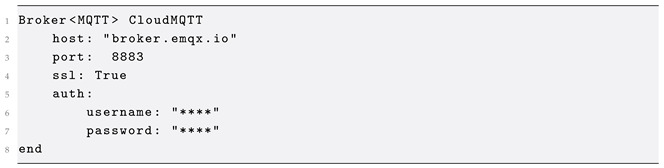


#### 2.2.3. Goal

**Figure 5:**
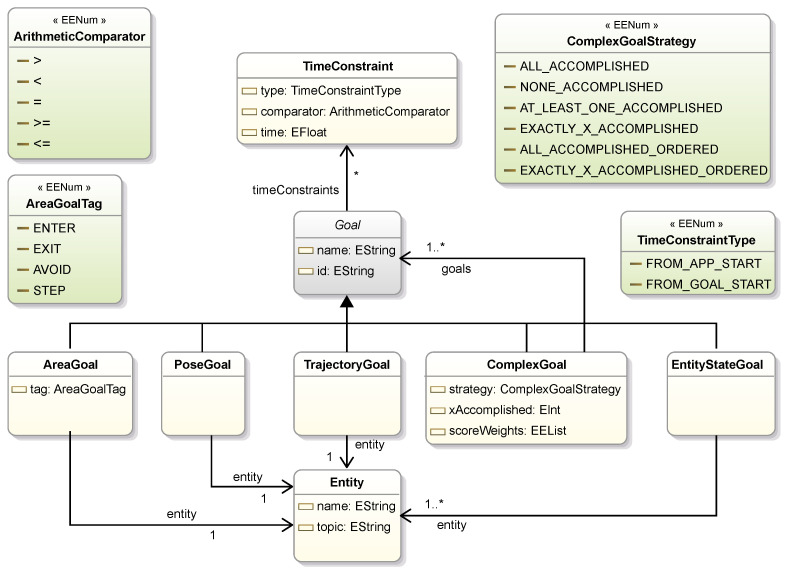
Goal meta-model. Modeling individual types of goals. ComplexGoal defines a concept for grouping goals with a single execution strategy (e.g., all accomplished ordered). The TimeConstraint concept introduces a way of applying time constraints to the goals.

In the proposed DSL, a Goal is defined for each expected behavior of an application or a system. As shown in Figure 5, GoalDSL defines five core super-classes of Goal: (1) EntityGoal, (2) AreaGoal, (3) PoseGoal, (4) TrajectoryGoal and (5) ComplexGoal. In Table 4, we include a detailed list of the supported classes of goals, along with a brief description of their functionality and use case.

**Figure 6:**
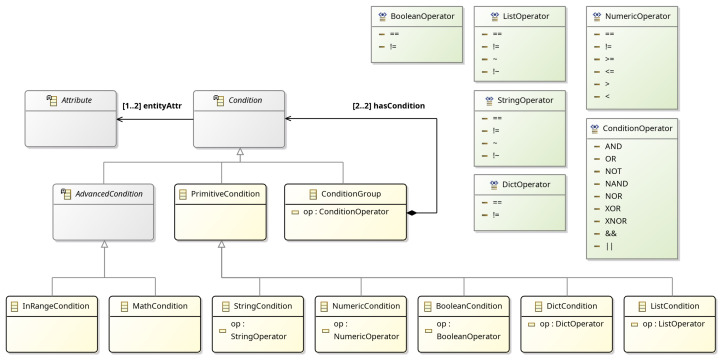
Condition meta-model.

The EntityStateChange and EntityStateCondition are generic classes, used to monitor changes in the state (the set of attributes) of an entity, or to apply conditional rules involving multiple entities, respectively. The latter introduces the Condition concept for applying advanced expressions. Conditions are divided into PrimitiveCondition(s), based on the data type of the parameters, and ConditionalGroup(s), used to define nested conditions. Furthermore, primitive condition classes have a reference to an attribute of an Entity, as shown in Figure 6. The language supports several conditional operators, including string, integer, float, boolean, dictionary, and list, as well as Python-style operators such as “is”, “in” and “has”. The AdvancedCondition class refers to advanced features and mathematical functions such as mean, max/min, and standard deviation of entity attributes (e.g., sensor values) in specific time frames, etc. Specifically, GoalDSL currently supports the following functions for attribute values: (a) mean, (b) std, (c) var, (d) min, (e) max, (f) in-range. Each Condition has a reference to at least one Attribute for applying the operator on either side (or both sides), as depicted below. In this example, the built-in mean advanced function and the InRangeCondition concept are used. Note the capability of the language to include attributes of multiple entities in a single condition.   


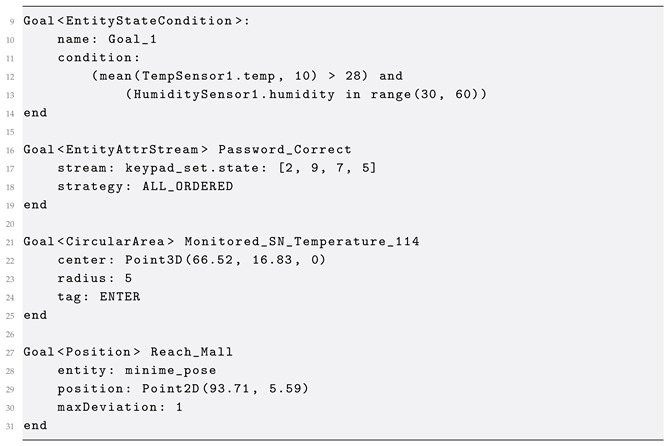


An AreaGoal is related to areas in the environment which have a meaning for an application, an example being the avoidance of specific areas. Pose goals (PoseGoal class) are those related to the position and orientation of a mobile thing, such as a robot. Trajectory-related goals (TrajectoryGoal class) are used to follow a trajectory, also used in mobile robot applications for navigation tasks.

In order to support complex scenarios, the ComplexGoal concept is introduced, which is defined by a list of goals and a strategy (ComplexGoalStrategy), such as for all/some/none goals to succeed. For example, by using complex goals, we can define a collection of goals, from which at least one has to be accomplished (Figure 5). In case of ordered execution strategies (*ALL_ACCOMPLISHED_ORDERED* and *EXACTLY_X_ACCOMPLISHED_ORDERED*) the execution of inner goals is sequential. Otherwise, the Scenario concept can be used to define the execution to either sequential or parallel. The Scenario concept defines a set of goals that are assigned to be executed for a specific scenario/application, and each model can define one or more Scenarios. Also, a Scenario can have reference to multiple goals, which are executed concurrently or in a sequential order. Score weights can be assigned to a ComplexGoal, similar to how these are set for a Scenario, as evident below.


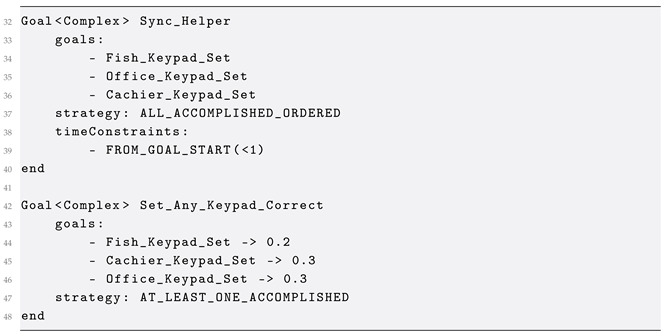


Finally, goals can have time constraints, like the maximum duration from the previous goal. For this reason, we introduce the TimeConstraint concept, which allows for the definition of time-related rules, within the body of a Goal. Each time constraint can measure time relative to either the start of the application (*FROM_APP_START* literal) or the start of the current goal (*FROM_GOAL_START* literal).

#### 2.2.4. Scenario

In GoalDSL, a Scenario serves as a fundamental construct for defining a collection of goals intended for a specific target or application. A key characteristic of a scenario is its ability to dictate the execution order of its constituent goals; the concurrent property determines whether these goals are validated simultaneously or in a sequential manner. For instance, a scenario might encompass multiple goals (e.g., Goal_1 through Goal_5), with their execution flow governed by this concurrent property, allowing for flexible and tailored behavior verification processes. The final score of a verification scenario in GoalDSL is calculated based on the in-model declaration of goal weights, based on the following equation:

(2) $$FinalScore=\vec{Gs}\cdot \vec{Ws}$$

The following two scenarios, *W3_T7* and *W3_T8*, exemplify distinct configurations for behavior verification with GoalDSL, showcasing both concurrent and sequential execution paradigms.


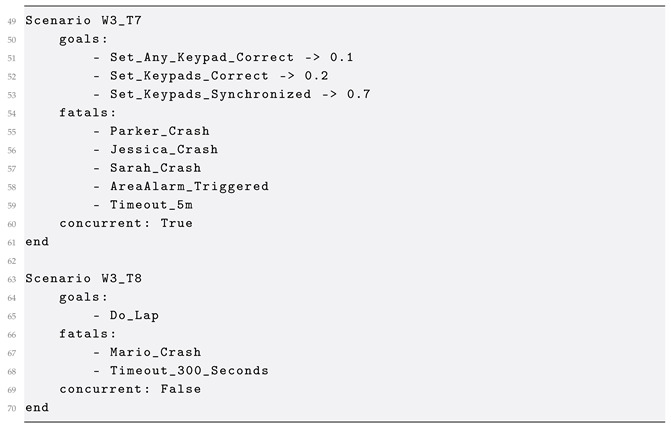


Scenario *W3_T7* defines a verification task focused on the correct and synchronized operation of keypad systems. Its primary goals include *Set_Any_Keypad_Correct, Set_Keypads_Correct*, and *Set_Keypads_Synchronized*, each assigned a weight (0.1, 0.2, and 0.7 respectively) to indicate their relative importance in achieving the scenario’s objective. The concurrent property signifies that these goals are concurrently evaluated, which is critical for verifying systems where multiple conditions or actions can occur in parallel. Furthermore, *W3_T7* incorporates several fatals (e.g., *Parker_Crash*, *Jessica_Crash*, *Sarah_Crash*, *AreaAlarm_Triggered*, *Timeout_5m*), which represent critical failure conditions. If any of these fatal events occur, the scenario immediately terminates, indicating a system failure. This configuration is particularly suitable for assessing the reliability of multi-component systems under various operational stresses.

In contrast, Scenario *W3_T8* presents a simpler, sequentially driven verification task. Its sole goal, *Do_Lap*, suggests a discrete action or process that needs to be completed. Similarly to *W3_T7*, this scenario also specifies fatals, namely *Mario_Crash* and *Timeout_300_Seconds*, which denote critical failure states that would lead to the immediate termination of the scenario. This type of sequential scenario is ideal for verifying the successful execution of specific, ordered tasks or processes where timing and completion without interruption are paramount, such as in robotic navigation or automated procedural operations.

The flexibility offered by the language, particularly the concurrent property and its strong tie to communication middleware, is pivotal for accurately modeling and verifying complex behaviors in diverse environments. By allowing goals to be executed either concurrently or sequentially, GoalDSL enables the precise representation of real-world interactions where actions might occur in parallel or demand a specific order. Furthermore, the explicit linkage of scenarios to specific message brokers facilitates the verification of systems distributed across various communication platforms, providing means for testing interactions between disparate entities and ensuring the holistic correctness of cyber–physical or digital twin systems. This design choice ultimately contributes to the methodology’s ability to automate verification processes for sophisticated, multi-component, microservice-based, and event-driven systems.

#### 2.2.5. RT Monitor

The RTMonitor concept in GoalDSL is essential for configuring the monitoring parameters of the runtime environment. It defines how compiled scenarios, managed by an executor, feed crucial runtime information—such as logs and event-related states—back into the system. This allows for comprehensive oversight of scenario execution and system/application behavior.

Specifically, it includes several key properties: (a) broker links to a defined communication middleware, ensuring that monitoring data is routed correctly, (b) *namespace* provides a URI prefix for constructing unique identifiers within the monitoring context, while (c) *eventTopic* and (d) *logsTopic* designate the specific message broker topics where real-time events and operational logs are published, respectively. An *extraAttr* property is also available for incorporating user-defined attributes, which can be leveraged by machine-to-machine (M2M) and model-to-text (M2T) transformations, as well as custom scripts, offering enhanced flexibility for advanced monitoring and analysis.


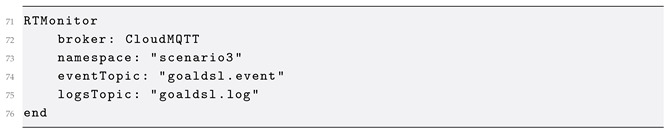


Next, Section 3 follows, presenting the experimental validation of the GoalDSL language, as it was used in a realistic setup, validating a large number of heterogeneous setups.

## 3. Experimental Validation

A series of comprehensive multi-discipline scenarios (experiments through a structured set of trials) were developed and executed within the context of an educational 3-week workshop setup to validate the effectiveness and expressiveness of the proposed DSL. The workshop took place in the context of the ECESCON conference (https://www.linkedin.com/company/ecescon/, accessed on 21 October 2025), offering a real-world validation of GoalDSL. The workshop had the form of a competition among teams, each of which was manned by 4 undergraduate electrical engineering students. Each team was provided with a number of tasks that spanned three distinct application domains: (a) classical algorithmic programming, (b) IoT automations, and (c) Robotics & CPS applications, further categorized by difficulty level (easy, medium, or high). This multifaceted validation approach showcases GoalDSL’s capacity to streamline the development of behavior verification processes for a wide range of IoT and CPS applications.

The competition was implemented online, using the LocSys platform [48], a comprehensive low-code web-based platform designed to host heterogeneous DSLs for CPS application development. The platform enables the integration of multiple DSLs, both graphical and textual, allowing developers to create, manage, and transform models across different aspects of complex domains. LocSys provides a unified interface through REST APIs, supporting standardized operators including validation, import, transformation, generation, and deployment that can be applied to all hosted DSL models. This architecture facilitates the creation of DSL pipelines, where models from one DSL can be systematically transformed into models of another DSL, ultimately producing deployable applications for smart environments and CPS.

**Figure 7:**
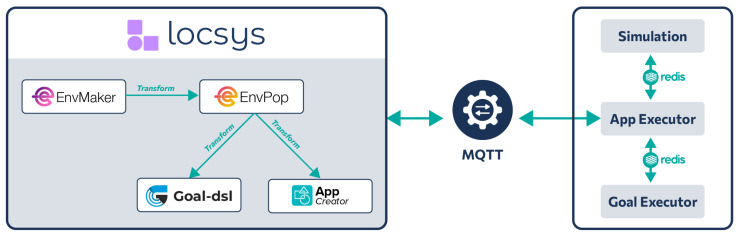
LocSys Integration Pipeline showing the multi-DSL workflow from environment design to behavioral verification.

As part of a comprehensive DSL pipeline that spans from environment design to application verification, GoalDSL was integrated into the LocSys ecosystem. Following LocSys’s established multi-DSL paradigm, GoalDSL operates through the systematic workflow illustrated in Figure 7.

**Figure 8:**
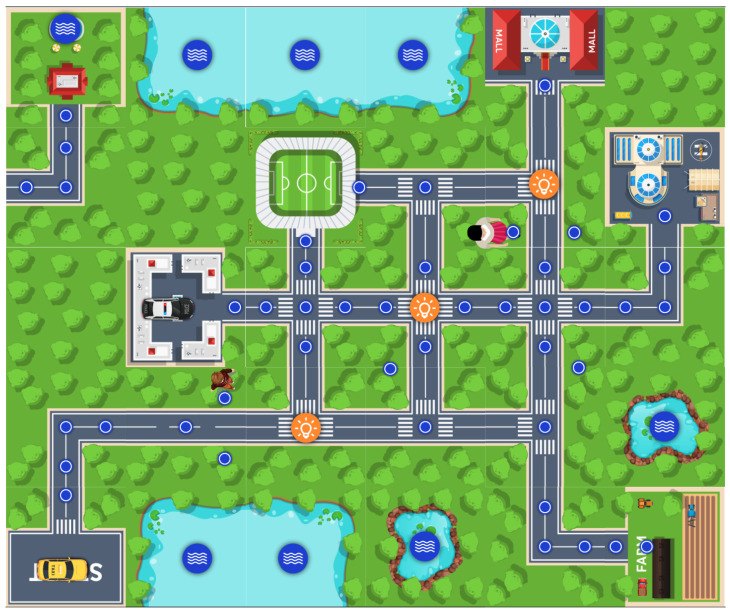
EnvPop model showing a smart city environment. Notation: Small blue dots—points of interest; Orange circles—smart lights; Blue circles—bodies of water; Cars—automated or manual smart vehicles; Child/Woman—actors with predefined trajectories.

The pipeline initiates with EnvMaker DSL, where the physical layout of the environment is declared for the IoT, CPS and Robotic-oriented scenarios. This model is then transformed into an EnvPop DSL model, where the environment becomes “smart” through the addition of sensors, actuators, and other IoT devices with their respective configurations, as shown in Figure 8.

**Figure 9:**
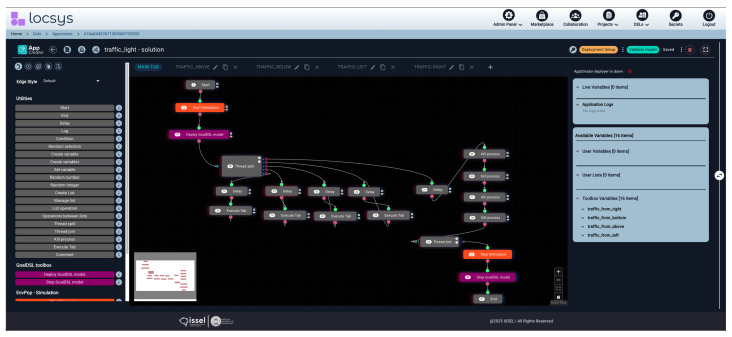
AppCreator model showing a flow-based application within the LocSys environment. The left column contains available toolboxes with their nodes, the middle part comprises a canvas where the application is created, and at the right part the available variables exist. Notation regarding the flow: Gray nodes—basic algorithmic functionality; Orange nodes—initiate and terminate the simulation environment; Purple nodes—initiate and terminate the GoalDSL validations.

After that, from the EnvPop model, the pipeline branches into two parallel paths. In the first path, the model transforms to AppCreator, a flow-based visual DSL where the competition participants were asked to design their solution’s logic through drag-and-drop operations (Figure 9). AppCreator provides several toolboxes, the most basic of which is Utilities, offering control flow elements (Start, End, Condition), data manipulation (Create variable, Set variable), operations (Delay, Log), and advanced features like thread management and list operations. Simultaneously, in the second path, the EnvPop model transforms to GoalDSL, where the competition organizers had defined goals, rules, and verification criteria for validating the behavior of the students’ solutions, according to the problem they were solving. Notably, AppCreator has integrated the GoalDSL deployment functionality, allowing users to deploy GoalDSL models directly from the visual programming interface, thus enabling the real-time validation of their solutions.

When the user initiates execution, LocSys orchestrates the deployment, where (a) the environment declared in EnvPop is simulated, generating realistic sensor data and responding to actuator commands, (b) the applications created by the students are deployed and interact with the simulator, managing the node-based workflows between connected blocks, and (c) the GoalDSL models created by the organizers are executed, monitoring the application behavior against the GoalDSL specifications, verifying that the solution for each specific scenario is correct, ultimately giving the participating teams the respective points.

### 3.1. Use Case Scenarios

A series of smart environments were specifically designed and developed for the competition, using LocSys no-code EnvMaker and EnvPop languages. These environments, serving as the operational backdrops for the diverse scenarios, were constructed to accurately simulate the complexities and interactions characteristic of real-world IoT and CPS deployments. By leveraging such environments, the study focuses on evaluating GoalDSL’s effectiveness in defining and verifying application behaviors, rather than being constrained by the technical overhead of environmental setup. This approach not only demonstrates the practical applicability of GoalDSL within low-code/no-code ecosystems but also highlights its potential to integrate seamlessly into broader development paradigms aimed at simplifying complex system design.

The following generated scenarios demonstrate how GoalDSL can indeed validate scenarios of different complexities and heterogeneous domains, bridging the gap between high-level validation requirements and executable system behaviors in digital environments (RQ1 & RQ2). GoalDSL models included in the experimental validation of this study are publicly available and hosted on GitHub (GoalDSL models: https://github.com/robotics-4-all/sfhmmy25-models, accessed on 21 October 2025).

#### 3.1.1. Programming: Foundational Logic and Data Manipulation

The programming tasks were designed to assess GoalDSL’s fundamental capabilities in handling computational logic and data manipulation, analogous to low-level programming constructs. The easy difficulty tasks included common programming challenges such as calculating the sum of numbers (1 to 100), performing list transformations (e.g., creating an *inverse_color* list for RGB values), calculating factorials, managing financial calculations with interest, segregating and summing odd/even numbers in a list, identifying specific divisible numbers, and determining the digit length of a number through iteration. Moving to medium difficulty, scenarios evaluated the DSL’s ability to process unique elements in a list, convert numbers to binary representation, implement sorting algorithms (e.g., Bubble Sort), identify specific number properties like Disarium numbers, and analyze sequences for gaps or ‘happy numbers’.

The high difficulty programming tasks delved into more complex computational problems, including calculating the average of prime numbers below a given value, checking for symmetric numbers, simplifying fractions iteratively, performing Taylor series expansions (e.g., sine calculation to 10 digits), identifying the longest alternating sequences of odd/even numbers in a list, and managing concurrent computations through multi-threading for tasks like sum calculation or variable manipulation under delays. These tasks collectively showcase GoalDSL’s support for foundational programming constructs and algorithmic implementation, validating its suitability for defining precise behavioral logic.

In the context of programming tasks, the *AppVariables* entity plays a pivotal role by enabling the direct integration of application variable state changes from the AppCreator environment into GoalDSL models. This crucial linkage allows for the definition of goals related to the application’s internal states, extending beyond simple variable values to complex data manipulation operations. For example, GoalDSL can define and verify objectives such as ensuring that elements are correctly added or removed from a list, that specific data transformations occur as expected, and that variables maintain particular values or ranges under various conditions. This capability is essential for verifying the correctness of the underlying application logic, ensuring that software components behave as intended.

Table 5, Table 6 and Table 7 describe these scenarios. To provide a measurement of the relative complexity of each verification task, we introduce a simple proxy for model size, S=E+G, where *E* is the number of entities and *G* is the number of goals defined in the model. This metric helps demonstrate the expressiveness and scalability of GoalDSL across tasks of varying scope (from simple to complex).

#### 3.1.2. IoT Applications: Sense–Plan–Act Automations

This category focuses on real-world scenarios within smart and digitized infrastructures, demonstrating the applicability of GoalDSL in IoT and cyber–physical applications. Examples include the following:

- Sensor Data Processing: Monitoring temperature sensors to process their values, calculating average temperatures from multiple sensors, and applying moving average filters to noisy CO_2_ sensor readings to detect human presence.
- Environmental control: Implementing logic for smart bulbs to react to changes in ambient brightness (turn on when it’s dark and turn off when it is light) or creating a controller for air conditioners to maintain a constant temperature in a house.
- Security and Access Control: Develop applications for keypad-operated doors that require specific passwords (e.g., the digits of the eighth perfect number) and define alert conditions based on combined sensor readings (e.g., high temperature and humidity).
- Complex Interactions: Designing traffic light controllers with timed green, orange, and red phases and developing camera-based applications that detect humans within specific sectors of a circle and recognize emotions (e.g., “happy”). A dynamic city illumination scenario based on autonomous car movement and alarm triggers further highlights the DSL’s capability in smart city applications.

Table 8 includes the IoT automation tasks, which involve interacting with various sensors and actuators to achieve specific goals.

#### 3.1.3. CPS Applications: Human–Robot and Multi-Robot Interaction

The smart automations with robot tasks involved scenarios with robotic agents in smart environments. These scenarios emphasized navigation, cooperative behaviors, and sensor-based decision-making. These challenges demonstrate GoalDSL’s ability to orchestrate complex interactions between robots and their environment, which include the following:

- Navigation and obstacle avoidance: This involves directing robots to specific points of interest (POIs) while avoiding environmental hazards, such as area alarms or linear alarms. More advanced navigation involves safely traversing while avoiding walls, cars, pedestrians, and red lights, using robot cameras to identify objects.
- Cooperative Robotics: Implementing tasks that require multiple robots to work together, such as Bonnie and Clyde cooperatively disabling linear alarms and reaching a destination.
- Sensor-Driven Tasks: Using robot cameras to read QR codes, identify unique words, and sort and interact with external switches. Another critical task involves using a heat sensor on a robot to verify temperature readings from fixed sensors in a nuclear power plant and identify “hijacked” sensors.
- Time-critical and emergency responses: Programming a robot to shut down switches in a nuclear power plant before reaching a critical temperature threshold (65 degrees Celsius, at which there is an explosion risk).
- Real-Time Control and Race Scenarios: Developing a rally robot that can complete laps within a time limit (300 s) while avoiding walls using distance sensors and relying solely on velocity set commands.
- Synchronized Multi-Robot Operations: Coordinating three robots to activate nine switches within a strict time limit (0.5-s deviation and five minutes overall) while avoiding area alarms and showcasing complex, synchronized actions.

Next, Table 9 includes the CPS tasks with robots, which were developed in the context of the experimental validation of the current study.

### 3.2. Discussion

To explicitly connect our experimental validation to the research questions posed in the introduction, Table 10 provides a summary matrix. It maps each RQ to the key indicators, evidence locations within the manuscript, and the principal conclusions drawn from our findings, thereby bridging our claims with the presented results.

The size of GoalDSL models, as documented in Table 5, Table 6, Table 7, Table 8 and Table 9, serves as a measurement for the complexity of the corresponding behavior verification scenarios. This metric is defined by the sum of the number of Entities and Goals utilized in each model (S = E + G). A larger model size directly correlates with a higher degree of complexity, indicating the usage of more conceptual instances. This trend is particularly evident in the CPS tasks involving multi-robot interaction (Table 9), which exhibit the largest model sizes. Accordingly, the progressive increase in model size across the programming, IoT automation, and CPS tasks reflects a gradual and quantifiable escalation in the complexity of the behavior verification processes for the applications.

While a formal quantitative comparison is considered an important future work key point of the current study, the qualitative benefits of GoalDSL’s high-level abstractions are logically evident when compared to its underlying internal DSL (Goalee) or GPLs. For instance, defining a complex goal like EntityStateCondition with time constraints in GoalDSL is a declarative, few-line task. Implementing the equivalent logic using a Python-based script would require manually coding message broker subscriptions, creating callback functions, managing state variables, and implementing timing loops—a process that is significantly more verbose, time-consuming, and prone to implementation errors. This level of abstraction directly supports our aim to accelerate development (RQ-1) by lowering the cognitive barrier for domain experts, allowing them to focus on behavioral and solution outcomes rather than implementation details (RQ-2).

The model size metric, S=E+G, as documented in Table 5, Table 6, Table 7, Table 8 and Table 9, serves as a proxy for the complexity of the behavior verification scenarios themselves. A larger model size directly correlates with a more intricate verification task, requiring a higher number of entities and behavioral goals to be specified. The clear trend of increasing *S* values from simple programming tasks (e.g., W1T3, S=2) to complex multi-robot CPS applications (e.g., W3T4, S=39) validates GoalDSL’s expressive range and its scalability to handle increasingly complex problems. This progressive increase reflects a quantifiable escalation in the inherent complexity of the application being verified. It is important to note that this metric is intended to demonstrate the DSL’s expressiveness across a wide range of problem complexity, not to make claims about development efficiency or quality, which require direct, user-based quantitative evaluation.

Specifically, the inclusion of classical programming tasks, ranging from basic arithmetic and list manipulation to complex algorithmic implementations and concurrent thread management, confirmed the DSL’s foundational expressiveness and its capability to capture granular computational logic. This ensures that even the most intricate application behaviors, often underpinning higher-level IoT and CPS functions, can be precisely defined and verified. Transitioning to IoT automations, the scenarios demonstrated GoalDSL’s direct applicability in managing sensor data, controlling smart devices, and orchestrating event-driven responses in smart environments. This domain highlighted how the DSL effectively abstracts away the complexities of diverse communication protocols and hardware interfaces, allowing users to focus on the desired automation logic itself. Finally, the Robotics & CPS application scenarios provided critical evidence of GoalDSL’s ability in orchestrating complex, multi-agent systems. These tasks, involving robot navigation, cooperative behaviors, and real-time interaction with physical environments, underscored the DSL’s unique strength in enabling high-level behavioral control and verification for intricate cyber-physical interactions. Together, these diverse validation cases prove GoalDSL’s versatility and its significant contribution to simplifying the development and behavior-level verification of IoT and CPS applications (RQ1).

The architectural design of GoalDSL further strengthens these capabilities. The explicit definition of goals and fatalities, along with the crucial concurrent property, allows for precise modeling of both parallel and sequential execution flows. This is critical for accurately representing real-world system dynamics. The RTMonitor mechanism provides a dedicated means for configuring runtime monitoring parameters, ensuring that crucial logs and event-related states are captured and fed back for comprehensive oversight of automation execution. Moreover, the flexible Entity concept extends beyond physical devices to seamlessly integrate with external consumer and producer cloud services (e.g., for storage, analysis, or data aggregation), enhancing the framework’s interoperability. The support for diverse external services and communication middleware (e.g., MQTT, AMQP, Redis), coupled with its protocol-agnostic architecture and context-aware design (RQ2), enables the construction of verification processes that can interact with multiple, heterogeneous environments, including both physical and virtual Digital Twin systems (RQ1).

While non-functional requirements (NFRs) like packet loss, out-of-order delivery, and jitter are currently handled by the underlying communication middleware (technology and protocol), this DSL will be enhanced to explicitly model these aspects. By doing so, the DSL will shift from merely relying on the communication layer to actively incorporating NFRs into the models. This will enable advanced pre- and post-analysis of system behavior, giving developers a more comprehensive understanding of the performance before deployment.

Furthermore, the current work is focused on providing pragmatic engineering support for CPS and IoT development. Its primary goal is to offer a high-level, domain-specific abstraction that simplifies the modeling and code generation of these complex systems. While the DSL is designed to enable behavioral verification of applications, the initial implementation and the current paper do not yet include a formal verification framework.

GoalDSL’s approach promotes increased productivity, minimizes the risk of implementation errors, and expands access for a broader range of end users to develop and verify applications in cutting-edge domains such as smart homes and smart industries. By automating the development of behavior verification processes, GoalDSL’s methodology enables developers to concentrate on the functional correctness and desired outcomes of their applications instead of being hindered by technical complexities. This establishes GoalDSL as a valuable tool for advancing the development and assurance of reliable IoT and CPS applications.

### 3.3. Limitations and Threats to Validity

In our study, we introduce a DSL for the development of verification processes for IoT-enabled CPS by adopting the low-code and DSL paradigms, thereby lowering complexity and the required time to verify the expected behavior of applications. While the experimental validation scenarios provide strong evidence of GoalDSL’s expressive power and utility, the current evaluation has several threats to validity that merit consideration.

Although experimental validation across 43 scenarios provides strong evidence of the expressive power of GoalDSL, the absence of a formal, quantitative baseline comparison is a primary threat to the validity of our claims regarding productivity. A next step would be to conduct a controlled experiment comparing development with the high-level GoalDSL to its underlying internal DSL (Goalee) and/or manual imperative scripting. To rigorously establish these benefits, the comparison should measure metrics such as LoC, task completion time, error rates, and cognitive load (e.g., via readability proxies or usability scales). The current study focused on demonstrating the DSL’s expressive range and establishing its methodology. However, we acknowledge that such empirical measurements are necessary to substantiate claims of productivity gains.

Furthermore, our reliance on self-reported feedback, without presenting formal quantitative usability metrics such as the System Usability Scale (SUS) [49,50], prevents us from empirically substantiating claims of productivity gains and reduced development burdens. We intend to perform a series of hands-on workshops to measure productivity gain for various user categories, which will include senior and junior professional developers, academic students, and citizen developers, as an extension of the current work to be published. To achieve proper productivity measurements, the SUS empirical evaluation test will be used, which is a highly robust and versatile approach for usability practitioners [51].

Another threat related to usability may exist concerning the adoption of a purely textual notation. While we selected a textual DSL for its precision and potential for AI-driven code generation [52], this design choice may pose a significant adoption barrier for the targeted domain experts or citizen developers who lack a programming background. These users often benefit from visual, drag-and-drop interfaces. While our grammar is designed for clarity, we have not empirically validated whether it is truly more accessible to this user group than a visual paradigm. Future work should directly compare the usability of our textual DSL against a visual counterpart for the same verification tasks.

A further limitation is the lack of a formal performance and scalability evaluation. The 43 use cases successfully validated the DSL’s expressive power, but they did not test the runtime performance of the generated source code under heavy load. In a real-world CPS deployment, the system might need to process thousands of events per second from numerous devices and concurrently evaluate hundreds of active verification goals. The current study does not provide data on metrics such as latency, resource consumption (CPU, memory), or throughput. It is currently unknown how the DSL scales and whether it could become a bottleneck in large-scale, time-critical applications. Performance benchmarking under simulated high-load conditions is considered a necessary next step in the evaluation of GoalDSL.

Additionally, while GoalDSL simplifies the creation of verification logic, the current work does not adequately address the debugging of this logic. When a verification goal fails, the system may report the failure, but it does not provide sophisticated debugging tools for the end-user. For a domain expert, it may be non-trivial to understand why a complex, multi-step goal failed (e.g., was it a logic error in the DSL script, a network-level event, or an unexpected device state?) The lack of dedicated debugging features, such as step-through execution, breakpoint-like features, or an execution trace visualizer, is a practical limitation that could hinder adoption by non-expert users who cannot easily troubleshoot their own verification scripts.

A noticeable limitation of the presented DSL is the lack of an integrated authorization layer. This is a significant drawback given the specific needs of CPS and the IoT, where security is a mandatory concern due to the increasing number of connected entities (devices, users, systems, etc.). To address this, the language needs to be extended to support access control mechanisms such as Role-Based Access Control (RBAC) or Attribute-Based Access Control (ABAC). These access control middleware would allow the definition of authorization rules directly within the DSL. By embedding this functionality, the DSL can ensure that interactions between devices, users, and systems are managed securely, mitigating risks associated with unauthorized access and data manipulation at runtime. This is a mandatory enhancement to make the DSL trustworthy for verifying the security aspects of IoT and CPS applications.

Finally, a noticeable limitation, as identified during our evaluation, is the current implementation’s focus on message-broker middleware for data integration. While our architecture is designed to be technology-agnostic, the existing version of the DSL only supports bindings for MQTT, AMQP, and Redis. This restricts the immediate verifiability of scenarios involving direct interactions with other common data sources, such as REST APIs, SQL, and NoSQL databases. To fully realize its portability, the grammar, as well as the M2T transformation and execution engine, must be extended. For instance, a REST binding would require extending the in-language supported data source definitions. The execution engine would then need to manage connection pooling, handle HTTP error codes (e.g., 404 Not Found, 503 Service Unavailable), and implement fallback or retry mechanisms. Similarly, SQL bindings would need to manage database connections and translate goal definitions into queries, while also being able to handle transaction integrity and connection errors.

## 4. Conclusions

In this work, we present a novel domain-specific language (DSL) designed to automate the development of behavior verification processes for complex IoT and CPS applications. To empirically validate the effectiveness and expressiveness of the proposed GoalDSL, a comprehensive set of tasks and scenarios (43 in total) was developed and executed within a 3-week educational workshop setup. These scenarios were carefully designed to cover three verticals: (a) classical programming, (b) IoT automations, and (c) Robotics & CPS applications. Furthermore, each domain was segmented into easy, medium, and high difficulty levels, thereby demonstrating the DSL’s inherent scalability and its capability to manage varying degrees of complexity. This multifaceted validation approach conclusively showcases GoalDSL’s significant capacity to streamline the definition and verification of behaviors across a broad spectrum of IoT and CPS applications, providing empirical evidence for its utility in real-world contexts and its potential to democratize access to advanced system development.

Future work will significantly expand the language’s capabilities by incorporating the spatial information of installed smart objects within predefined environments. This enhancement will allow for the creation of models that can directly reference the physical locations of devices. This will facilitate advanced verification processes against spatial restrictions and topological constraints. In addition, tools such as LocSys are expected to play a pivotal role by empowering users to quickly design and automatically generate digital twins of relevant smart home environments (or others) from ready-to-use components on a canvas. As detailed in earlier sections, these virtual environments comprise a diverse array of smart objects (e.g., sensors, actuators, and robots) that interact through common communication middleware. They will serve as rich and realistic testbeds for spatially aware behavior verification processes defined by our domain-specific language (DSL). This integration will further bridge the gap between abstract behavioral logic and the physical realities of complex cyber-physical systems.

A parallel line of future work will focus on extending the execution framework with bindings for other data sources, including REST APIs and databases, to broaden the DSL’s interoperability, as discussed in Section 3.3.

Furthermore, a promising direction for future studies is to conduct a formal feature ablation study. Such a study could quantitatively measure the impact of core semantics, such as time constraints and concurrency, by analyzing the increase in false positives and non-expressible scenarios when these features are disabled. This would empirically validate their necessity in the CPS domain.

Finally, building upon the previously identified limitations of the study, we will conduct a formal empirical study to quantitatively measure productivity gains, structured as a series of hands-on workshops involving diverse user groups (professional developers, academic students, and citizen developers). The study will feature a direct comparison between three development approaches: (1) using the high-level GoalDSL, (2) using the Python-based internal DSL (Goalee), and (3) manual scripting from scratch. To achieve a robust evaluation, we will employ the Goal-Question-Metric (GQM) approach [53] to systematically measure differences in key indicators such as LoC, development time, and task success rates. Furthermore, we will incorporate the SUS [49,50] to perform empirical evaluation [51] on user-perceived usability and cognitive experience, providing a comprehensive assessment of the practical benefits of our high-level, domain-specific approach.

## Figures and Tables

**Table 1:** Systematic Comparison of IoT/CPS DSLs and Frameworks.

DSL/Framework	Primary Focus	Target User	Core Paradigm	Verification Support	Technology Agnostic
ThingML [28]	Application Development	Developers	Component & State-based	Model consistency checks	High
SmartHomeML [31]	Application Generation	Developers	Rule-based	None (Generates code)	Low (Web-centric)
FRASAD [30]	Application Development	Developers	Rule-based	None (Focus on design)	Moderate
CyprIoT [42]	Application Development	Developers	Rule-based	Model-level only	Low (Web-centric)
Triton [32]	Embedded Code Generation	Developers	Task & Constraint-based	Code-level constraints	Low (Embedded focus)
Andova et al. [35]	System Specification	Formal Methods Experts	Process Algebra	Formal (LTS Transformation)	High
Monitor-IoT [36]	System Specification and monitoring	Developers	Task based	Model-level only	Low (MAPE-K specific)
aDSL [37]	System Specification and Verification	Developers	Model-level only	Formal	High
ETL [39]	System Specification and Validation	Developers	Rule-based	Runtime	Low (MODELICA specific)
Our Approach (GoalDSL)	Behavior Verification	Domain Experts/End-Users	Goal-driven	Runtime Goal Checking	High (Protocol-agnostic)

**Table 2:** GoalDSL REST API endpoints.

Endpoint	Verb	Description
/model	POST	Store Model in model repository.
/model/{model_id}	GET, PUT, DELETE	Retrieve/Update/Remove model from the repository, given the model id.
/model/validate	POST	Perform validation on input model file.
/model/{model_id}/validate	POST	Perform validation on previously uploaded model, given the id.
/model/execute	POST	Dynamically build and execute verification processes from input model file.
/model/{model_id}/execute	POST	Dynamically build and execute verification processes from previously uploaded model.
/model/codegen/goalee	POST	Perform code generation of verification processes from input model file.
/model/codegen/ventities	POST	Perform code generation of virtual entities from input model file.
/model/{model_id}/codegen/ventities	POST	Perform code generation of virtual entities from a previously uploaded model.
/model/{model_id}/codegen/goalee	POST	Perform code generation of verification processes from a previously uploaded model.
/user/{username}/model/{model_id}	GET	Retrieve a previously uploaded model from the repository.

**Table 3:** Metrics for evaluating the size of GoalDSL meta-models.

Metamodel	Entities	Relations	Types	C	A	E	R	S
GoalDSLRootMM	8	6	4	11	2	1	4	18
GoalMM	8	6	4	11	2	1	4	18
ConditionMM	8	10	1	13	4	6	6	19
DataTypeMM	14	4	0	14	4	0	0	18
EntityMM	12	3	0	11	2	1	1	15
CommunicationMM	9	3	2	11	3	0	0	14

**Table 4:** Supported goal classes with brief description of their functionality.

Class Name	Description
EntityGoal	
EntityStateChange	Goal succeeds when the state of a reference Entity is changed.
EntityStateCondition	Applies conditions on reference Entity attributes.
EntityAttrStreamGoal	Work with a pre-defined streams of entity attributes.
EntityStateTransitionGoal	Define a state transition to monitor, given previous and next states.
**AreaGoal**	
RectangleArea	A rectangle area defined by the center point and a radius.
PolylineArea	A polyline area defined by a list of Points [$\left(x_{i},y_{i},z_{i}\right)$]
MovingArea	This type of Goal can be used for mobile objects, such as robots, to check for area reach and avoidance.
CircularArea	A circular area defined by a center point and a radius.
CrossStraightLine	A straight line in the environment defined by a starting point and a finish point.
**PoseGoal**	
Position	Reach a specific position in 3D space.
Orientation	Reach a specific orientation in 3D space.
Pose	Reach a specific pose (orientation, position) in 3D space.
**TrajectoryGoal**	
StraightLineTrajectory	Follow a straight line, defined by: (a) starting point, (b) finish point, (c) maxDeviation.
CurveTrajectoryGoal	Follow a curve trajectory, defined by: (a) starting point, (b) finish point, (c) maxDeviation, (d) curvature.
WaypointTrajectory	A custom trajectory goal defined by list of points and a maximum deviation.

**Table 5:** Programming Tasks—Easy Difficulty.

Task Name (Codename)	Task Description	Model Size (S)
Sum 1 to 100 (W1T1)	Calculate the sum of the numbers from 1 to 100 and store it in the variable sum. In the loop you will implement, use the variable i.	1E+6G=7
Inverse Color List (W1T2)	Create a list of values [45, 89, 113] representing an RGB color. Create a list named inverse_color, which contains the negative color.	1E+2G=3
Factorial and Capital (W1T3)	Calculate the 11 factorial and store it in the variable x. Use variable i for the loop. If our initial capital is 1000 euros and we have 1% interest each month, calculate our capital at the end of the 2nd year and save it in the capital variable.	1E+1G=2
Odd/Even Sum List (W1T4)	Create a list x with elements [6, 9, 14, 56, 99, 145, 198, 3, 93, 22, 245, 798]. Create a list named result, where the first element is the sum of all odd numbers and the second is the sum of all even numbers from x.	1E+4G=5
Divisible Number (W1T5)	Create variables a = 148 and b = 43. Calculate the number greater than a and b that is exactly divisible by b. Store it in variable c.	1E+3G=4
Digit Length (W1T6)	Create a variable x with value 5,819,546. Calculate its length in digits and store the result in the length variable using iteration.	1E+7G=8

**Table 6:** Programming Tasks—Medium Difficulty.

Task Name (Codename)	Task Description	Model Size (S)
Unique Sorted List (W1T7)	Create list x with elements [9, 5, 7, 1, 0, 9, 5, 3, 7, 6, 2, 5]. Compute a list named final, containing unique elements of x in ascending order.	1E+7G=8
Binary Conversion (W1T8)	Create variable number = 151. Convert it to its binary equivalent and store the result in the variable binary.	1E+6G=7
Bubble Sort Desc (W1T9)	Implement the bubblesort algorithm for a list with initial elements [13, 9, 67, 93, 45, 1, 85, 64, 0, 44], sorting it in descending order.	1E+1G=2
Disarium Check (W1T10)	For variable xx = 2,646,798, store 1 in variable result if it’s a Disarium number (sum of digits raised to position power equals the number), else store 0.	1E+14G=15
Sequence Gaps (W1T11)	Given the lists x = [9, 5, 7, 1, 3] and y = [8, 2, 6, 4], store 1 in result if combining and sorting creates a sequence without gaps (e.g., 4, 5, 6, 6, 7, 8 has no gaps), else store 0. Sort the combined list as xy.	1E+7G=8
Happy Number (W1T12)	For variable x = 89,456, store 1 in the variable result if it’s a “happy number” (sum of squares of digits, iterated, eventually reaches 1), else store 0. Store intermediate sums in tmp_sum.	1E+1G=2

**Table 7:** Programming Tasks—High Difficulty.

Task Name (Codename)	Task Description	Model Size (S)
Prime Average (W1T13)	For variable x = 78, calculate the average of prime numbers less than x. Store the result in the variable result, and the prime numbers in primes. Assume 2 and 3 are known primes.	1E+4G=5
Symmetric Numbers (W1T14)	For x = 9,678,769 and y = 5,566,216,655, store 1 in x_result and y_result if the number is symmetric (reads same forwards/backwards), else store 0.	1E+8G=9
Simplify Fraction (W1T15)	Given ‘a’ (960) and ‘b’ (320) forming a fraction a/b, calculate its simplified form c/d by iteratively checking common divisors.	1E+9G=10
Sine Taylor Series (W1T16)	Calculate the sine of 0.5999 radians to 10 decimal places using the Taylor series expansion. Store intermediate sums in ‘temp’, final result in ‘sin’.	1E+6G=7
Alternating Sequence (W1T17)	For list ‘x’ [3, 7, 4, 6, 8, 2, 3, 1, 5, 3, 2, 1, 6, 1, 5, 7, 8, 2, 3, 6, 8, 2], find the longest sequence of alternating odd and even numbers and store it in ‘result’.	1E+9G=10
Threaded Sum (W1T18)	Calculate the sum from 1 to 300 using 3 threads, each summing 100 numbers. Store individual sums in *sum_1*, *sum_2*, *sum_3* and total in *total_sum*.	1E+2G=3
Concurrent Variable (W1T19)	For variable ‘x’ (1542), use two threads: one adds 10 (0.5 s delay), another divides by 2 (0.34 s delay). If ‘x’ reaches 7, set ‘y’ to 1; if not after 100 assignments, set ‘y’ to −1.	1E+4G=5

**Table 8:** IoT Automations Tasks.

Task Name (Codename)	Task Description	Model Size (S)
Double Sensor Data (W2T1)	For ‘my_sensor’ (temp sensor generating data every second), store twice the received data in ‘double_sensor_data’ for 30 s.	2E+4G=6
Average Temperature (W2T2)	Calculate the average temperature from three house temperature sensors every second for 30 s, storing it in ‘average’.	4E+7G=11
Smart Bulbs Control (W2T3)	Control 3 smart bulbs: turn on when ambient brightness is <30% and off when >70%. Ensure at least one on/off cycle.	7E+10G=17
Temp-Humidity Alert (W2T4)	Set ‘alert’ to 1 if any of two temp sensors measures >27 °C AND humidity sensor measures >80% simultaneously, checking for 30 s.	8E+5G=13
Keypad Door (W2T5)	Implement a keypad door where the password is the digits of the 8th perfect number (2305843008139952128).	2E+1G=3
Filtered Temp Counter (W2T6)	Apply a simple moving average filter to spontaneous temperature values. Count how many times the filtered temperature value transitions above 23.8 degrees Celsius in 2 min.	2E+7G=9
Sinusoidal Keypad (W2T7)	Unlock a door via a keypad where the code is the maximum temperature (rounded) from a sinusoidal temp sensor followed by the minimum humidity (rounded) from a sinusoidal humidity sensor.	2E+1G=3
Traffic Light Ctrl (W2T8)	Implement a traffic light controller for an intersection: green for 2 s (RGB 0,255,0), orange for 1 s (RGB 255,165,0), red for 3 s (RGB 255,0,0).	9E+27G=36
Pan-Tilt Human Detect (W2T9)	Control a pan-tilt camera to detect people. Calculate how many of 12 sectors (30-degree each) contain a person and store in ‘human’. Also count “happy” people in ‘happy’. Camera FOV is 90 degrees.	2E+1G=3
CO_2_ Human Counter (W2T10)	Use a CO_2_ sensor (with simple moving average filter) to detect humans; increment ‘humans’ by 1 per detection. Terminate after 2.5 min.	6E+1G=7
AC Temperature Ctrl (W2T11)	Implement a controller for two air-conditioners to maintain a point of interest at 22.5 (±0.5 degrees) for 20 s, adapting to changing outdoor temperature.	6E+1G=7
Angry Human Angle (W2T12)	Control a pan-tilt camera to find the angle (in radians, to nearest 0.5 degrees) of an angry human in a nearby room, starting search from −90 degrees. Camera FOV is 90 degrees.	6E+2G=8
Multi-Camera Tracking (W2T13)	Use 4 pan-tilt cameras (camera_1/ef_panTilt_39, camera_2/ef_panTilt_40, camera_3/ef_panTilt_41, camera_4/ef_panTilt_38) to track a man moving around a room, ensuring he remains in at least one camera’s FOV using detections.	6E+5G=11
Smart City Illumination (W2T14)	Dynamically illuminate a smart city: when an autonomous car passes a linear alarm or enters an area alarm, switch on the relevant street lighting for 10 s.	18E+15G=33

**Table 9:** CPS tasks with Multi-Robot Interaction.

Task Name (Codename)	Task Description	Model Size (S)
Mall Navigation (W3T1)	Drive robot *2fast4you* to the Mall without triggering any area alarm.	6E+3G=9
Mall Navigation (W3T2)	Drive robot *minime* to the Mall, without triggering a linear alarm and without using *Go to POI* commands.	12E+4G=16
Cooperative Alarm Disable (W3T3)	Move robots *bonnie* and *clyde* cooperatively to disable linear alarms (only deactivated near a robot) and both reach the Mall.	13E+5G=18
Safe Robot Navigation (W3T4)	Drive robot *Gerard* safely to the Mall, avoiding walls, other cars, pedestrians, and red lights, using its camera for identification (20% detection failure probability).	22E+17G=39
QR Code Collect (W3T5)	Use robot *gn_robot_69* to visit all QR codes, identify unique words, add them to a sorted list, then toggle *freedom* switch using the first and fourth sorted words. Robot lights must be on.	13E+23G=36
Faulty Sensor Check (W3T6)	Drive robot *whitehat* (with heat sensor) to 6 nuclear power plant temp sensors, check consistency with robot’s measurements, increment *highjacked* for each faulty sensor (man-in-the-middle).	11E+13G=24
Emergency Shutdown (W3T7)	Send a robot to shut down three main switches in a nuclear power plant before central temperature sensor reads 65 degrees Celsius and an explosion occurs.	16E+11G=27
Rally Race (W3T8)	Drive rally robot *mario* for two laps of a race track within 300 s, avoiding walls using distance sensors (twice per second readings), only using ‘Velocity set’ command.	10E+9G=19
Cooperative Supermarket Access (W3T9)	Coordinate ‘Sarah’, ‘Jessica’, and ‘Parker’ robots to simultaneously (0.5 s deviation) give commands (‘nine’, ‘tango’, ‘charlie’) to three switches within 5 min, avoiding area alarms.	26E+12G=38

**Table 10:** Research Questions to Evidence Mapping Matrix.

RQ	Key Indicator	Evidence Location	Key Takeaway
RQ-1: Accelerate development?	High-level Abstractions & Automation	Section 2.2 (DSL Concepts), Section 3 (LocSys Pipeline, Figure 7, Figure 8 and Figure 9)	The goal-driven DSL and automated code generation pipeline accelerate development by abstracting away low-level technical complexities.
	Expressiveness Across Diverse Use Cases	Section 3.1 (Table 5, Table 6, Table 7, Table 8 and Table 9)	The successful and concise modeling of 43 diverse scenarios demonstrates the DSL’s capability for rapid application and verification compared to manual, from-scratch implementation using GPLs.
RQ-2: Abstracts domain complexity?	Minimized Technical Knowledge Requirement	Table 1, Section 2.2.3 (Goal Examples), Section 3.2 (Workshop Context)	The declarative, human-readable syntax of the language allows domain experts to focus on the “what” (behavioral outcomes) rather than the “how”(implementation), lowering the technical barrier.
	Framework-, Protocol-, & Device-Agnostic	Section 2.2.1 (Entity), Section 2.2.2 (Broker, Figure 4), Section 3.2 (Discussion)	The architectural approach of decoupling abstract entities from concrete communication bindings (e.g., MQTT and AMQP brokers) ensures protocol and device neutrality.

## Data Availability

GoalDSL models included in the context of the experimental validation of this study are publicly available at https://github.com/robotics-4-all/sfhmmy25-models/tree/main/goaldsl, accessed on 21 October 2025.

## Abbreviations

The following abbreviations are used in this manuscript:

IoT	Internet of Things
CPS	Cyber–Physical Systems
MDE	Model-Driven Engineering
MDD	Model-Driven Development
DSL	Domain-Specific Language
GPL	General-Purpose Language
API	Application Interface
URI	Uniform Resource Identifier
